# Exploring Continuous Pressure Monitoring to Inform Decisions for Pressure Injuries in the Community: Secondary Analysis Using a Mobility and Pressure Exposure Algorithm

**DOI:** 10.1111/iwj.70896

**Published:** 2026-04-12

**Authors:** Nicci Aylward‐Wotton, Silvia Caggiari, Bridie Kent, Liudi Jiang, Rashedul Hoque, Peter R. Worsley

**Affiliations:** ^1^ Skin Sensing Research Group, School of Health Sciences University of Southampton Southampton UK; ^2^ Cornwall Partnership NHS Foundation Trust, Carew House Beacon Technology Park Bodmin UK; ^3^ Faculty of HealthSouth‐West Clinical Schools University of Plymouth Plymouth UK; ^4^ School of Biomedical Sciences, Faculty of Biological Sciences University of Leeds Leeds UK; ^5^ School of Nursing and Midwifery University of Plymouth Plymouth UK; ^6^ Faculty of Engineering and Physical Sciences University of Southampton Southampton UK

**Keywords:** continuous pressure monitoring, exposure threshold, intelligent algorithm, pressure injuries, PROMISE

## Abstract

Frailty in community‐dwelling individuals often leads to prolonged periods in bed or sitting, increasing their risk of pressure injury development. The Quality Improvement project ‘Pressure Reduction through cOntinuous Monitoring In the community SEtting’ (PROMISE) implemented the use of continuous pressure monitoring (CPM) to inform interventions. A secondary analysis of PROMISE data involving 17 patients was examined before and after the intervention. A novel algorithm using duration and magnitude of pressure signatures at the buttock area was estimated from the CPM data and an algorithm based on the sigmoid relationship between pressure and time was used to categorise risk pre‐ to post‐intervention. The CPM intervention helped inform changes in support surface, posture and mobility advice. Duration and magnitude of pressure signatures revealed a high degree of inter‐subject variability. At baseline 35% of (6/17) patients spent prolonged periods with potentially harmful interface pressures (high to very high exposure). Trends of improvements post‐PROMISE intervention were observed, with 24% (4/17) in these higher exposure categories. This study demonstrated how CPM could be used to inform interventions for individuals living with pressure injuries in the community. An algorithm was used to understand trends in posture, mobility, and pressure exposure, showing some improvement pre‐ to post‐intervention.

## Key Points

- Frail community patients are at high risk of pressure injuries due to prolonged sitting/lying, during the PROMISE study it emerged that continuous pressure monitoring (CPM) guided personalised interventions.
- The present study performed a retrospective evaluation of continuous pressure monitoring data from PROMISE using a refined algorithm to predict posture, mobility and pressure exposure.
- The algorithm demonstrated the potential to stratify levels of risk in community patients and was sensitive to detect changes pre‐ and post‐ interventions, with a reduction in high exposure levels.
- CPM and associated algorithms could improve pressure injury management in community care, however key human factor and economic evaluations are required to support implementation.

## 1 Introduction

Pressure injuries/ulcers are localised damage to the skin and/or underlying tissue, usually over bony prominences or related to medical or other devices, resulting from prolonged pressure or pressure in combination with shear [1]. They are classified from Category 1 to 4, depending on the level of severity, representing serious chronic disabling conditions [2], which cause pain and distress and are a source of infection.

In the UK, over 700 000 people are affected by pressure ulcers each year [3]. Many of these occur in the community setting and become long‐term, non‐healing wounds, leading to significant discomfort. The risk of PUs is reduced by providing cushions and mattresses suitable for patients who are immobile, changing their posture infrequently [4]. The total cost in the UK is estimated between £1.4 and £2.1 billion per year, which represents approximately 4% of total NHS expenditure [5]. On average, each community patient costs approximately £1400 p.a. for Category 1 and > £8500 for Category 4 PUs, with the majority of this cost represented by nurse time [6]. Unlike patients in hospital, those in the community spend a relatively small amount of time with healthcare practitioners, with many care episodes cut short [7]. Therefore, strategies are required to ensure they remain protected from the worsening of existing pressure damage or the development of new skin damage. For this reason, community patients require an addition to a risk assessment which would be needed to outline the patient's risks and develop patient‐specific strategies [1]. These involve pressure‐relieving equipment, mattress type, mobility aids and nutrition, and should be monitored to ensure they meet patients' needs [4].

Technologies have been seen to act as a potential adjunct to clinical decision‐making through monitoring devices and biofeedback [8]. One of the most common technologies used in care settings has been pressure mapping. However, these are typically implemented to provide snapshots of seating or lying pressure, limiting their ability to monitor over time [9]. In recent years this technology has been modified to record over prolonged periods. The Quality Improvement (QI) project ‘Pressure Reduction through cOntinuous Monitoring In the community SEtting’ (PROMISE) used continuous pressure monitoring (CPM) sensing technology in individual community residents [10]. These sensing arrays, which continuously measure the distribution of pressure, visually identify the areas of the body under sustained pressure on a display monitor and can inform adjustments to support surface selection and postures [11]. They were also used to empower patients, carers and clinicians to improve their understanding of pressure risk and promote shared decision‐making. This approach differs from previous studies which have used indicators such as mean pressure to make comparisons between patients [12].

Clinical applicability of different sensing technologies including pressure monitoring systems has been discussed in a recent review [8], highlighting lack of innovation of the current commercial systems in detecting posture and mobility in bed. A recent study used changes in the correlation coefficient of pressure values above 40 mmHg between consecutive time points to determine changes in posture [13]. The authors developed a more sophisticated algorithm and a secondary analysis was conducted on the continuous pressure data recorded during patients' initial assessment, to evaluate their posture, mobility, and pressure exposure [9, 14, 15]. Findings showed those who had acquired a pressure injury at the time of initial monitoring exhibited trends which exposed their skin to prolonged high pressures during static postures [15], highlighting that many individuals in community settings often experience prolonged periods of static lying postures. This may reflect the personal circumstance of health or social care support, leading to an inability to regularly change positions [16]. There were, however, limitations in the approach, where pressure gradients over the surface of the bed were assessed, rather than specific sites over the pressure injury for example, buttock.

The authors have previously reported on 22 patients from the PROMISE project who had pressure injuries and were initially assessed through continuous pressure monitoring [15]. From this first analysis, it emerged that most of the patients had prolonged periods of immobility and high pressure exposure. However, this was limited to an initial assessment period, and an algorithm that looked at gross mobility and whole body pressure exposures. There is a need to assess how the CPM technology and associated algorithms can detect changes before and after an intervention and whether exposure to prolonged harmful loads over the site of the pressure injury (i.e., buttocks) could be changed through clinical interventions. This current secondary analysis involved 17 patients from which CPM data was collected both pre‐ and post‐ PROMISE intervention. The aim of this study is to demonstrate how CPM could be used to understand trends in posture, mobility and pressure exposure at the buttocks before and after a tailor pressure injury prevention programme. The present study will report on the clinical presentation of the community patients, detail their interventions and report on the changes in mobility and pressure exposure pre‐ and post‐intervention.

## 2 Methods

This study represents a secondary analysis of the QI PROMISE project, which obtained institutional ethical approval (ERGO 80625. A1). During the PROMISE study, 105 community residents (individuals in private, residential and nursing homes) were recruited from four regional healthcare providers based in Southwest England (four community trial sites). Individuals were eligible if they were deemed at high risk of PU development, had existing pressure injuries or were reluctant or unable to use their allocated pressure relieving equipment. Informed consent was obtained from each patient prior to the monitoring period, or where appropriate consent via a consultee. Data collection was carried out in accordance with The Code of Ethics of the World Medical Association (Declaration of Helsinki) for experiments involving humans. This secondary analysis was performed on 17 out of the original 22 patients previously analysed for posture and mobility [15], with 5 data sets omitted due to loss of post intervention data.

### 2.1 Continuous Pressure Monitoring (CPM) Technology

A commercial system (ForesitePT, Xsensor, Canada) was used in the PROMISE study. The ForeSite PT is a bed sensing array and consists of a fitted mattress cover embedded with 5556 sensor cells, over a surface area of 762 × 1880 mm and spatial resolution of 15.9 mm. The system continuously recorded interface pressure values with a sampling frequency of 1 Hz.

Its performance characteristics following mechanical compression loading increments with a dual hemispherical buttock shaped indenter were evaluated in a recent study [17], demonstrating robustness in the pressure readings.

Sensor arrays were used in each community patient recruited to the study, placed on top of the mattress, underneath a bed sheet, with the dedicated monitor showing an image of the pressure distribution. The systems were cleaned between patient usage as per the infection prevention standards of the healthcare institution.

### 2.2 PROMISE Intervention

**FIGURE 1:**
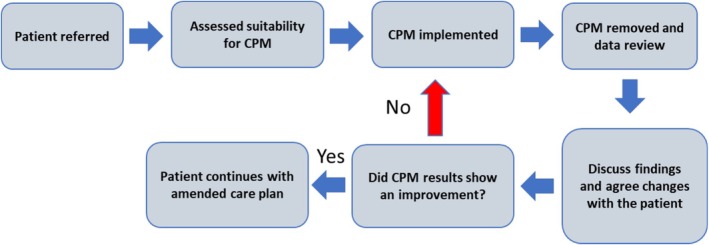
Different phases of the PROMISE intervention.

The CPM technology was implemented in community residents with pressure injuries and patients who were reluctant to use pressure relieving equipment or where patients found their current support surface to be uncomfortable. Patients were monitored between 5 h and 3 days for an initial period of assessment, with clinical interventions provided in line with recommended national and international guidelines [18, 19]. The data from the CPM were downloaded and shared between healthcare professionals, patients and carers to improve shared understanding and decision‐making. This considered the holistic context including social factors, patient preference and the environmental factors relevant to the setting (patients' home, residential care, or long‐term care facility). An equipment and repositioning review was then conducted to optimise the pressure redistribution whilst promoting good posture and mobility, with periods of CPM assessment to inform practice. Figure 1 depicts the phases of the PROMISE intervention. Clinicians also identified the interface pressure values (Peak Pressure, Peak Pressure index, and Pressure Gradient) over the same site of the pressure injury on the patient's body [20]. Once a solution was identified, a final CPM assessment was conducted to assess changes from pre‐intervention.

### 2.3 Clinical Outcome Measures

The PROMISE QI study assessed and monitored several clinical outcomes to determine whether CPM informed interventions aided clinical decision making and pressure injury prevention/healing. These included:

- Pressure injury healing rates.
- Time to healing following intervention.
- Level of carer support.
- Patient mobility.
- Bed‐bound (BB) status.
- Sitting‐out (SO) time.
- Use of a wheelchair.
- Prevention.

Demographic data were collected for each patient, including body mass index (BMI), primary diagnosis, age, gender, and the location of the patient in which CPM was used. The number of CPM sessions delivered to each patient was also recorded (Table 1). The majority of patients had a high frailty index and either formal or informal carer support. Pressure ulcer healing was determined via clinicians measuring the wound length × width × depth on each visit over the period of the PROMISE study. This was confirmed by photos at the end of the study and reviewed by three tissue viability specialist nurses.

**TABLE 1:** Pressure injury category and support surface type pre‐ and post‐intervention.

Patient No.	Age	Sex	Patient Location	BMI	Frailty	PMH	PU Cat—pre intervention	PU location	Mattress type—pre intervention	PU Cat—post intervention	Mattress type—post intervention	Carer involvement
1	74	F	Home	46.7	6	Osteo Arthritis	Cat 2	Buttock	Dynamic Air Overlay	Healed	Dynamic Air Overlay	1 time per day
2	92	F	Home	24.3	6	Dementia	Cat 2	Buttock	Dynamic Air Overlay	Healed	Static Air Overlay	Continuous
5	66	M	Home	32.9	7	MS	Cat 2	Buttock	Static Foam Mattress	Healed	Static Air Overlay	4 times per day
6	66	F	Home	32.0	6	SCI	Cat 2	Buttock	Own Mattress	Healed	Static Air Overlay	None
7	52	F	Home	25.5	6	MS	Unstageable	Hip	Static air Overlay	Healed	Air Floatation	Continuous
8	86	F	Home	15.1	8	CVE/Dementia	Unstageable	Sacrum	Dynamic Air Mattress	Unstageable	Dynamic Air Mattress	4 times per day
9	82	F	Home	37.5	6	CVE	Cat 2	Buttock	Dynamic Air Overlay	Healed	Air Floatation	4 times per day
10	30	M	Home	22.5	7	Spina Bifida	Cat 4	Spine	Dynamic Air Mattress	Healed	Dynamic Air Mattress + Turn Device	Continuous
11	65	F	Home	31.0	7	MS	Cat 2	Buttock	Static air Overlay	Healed	Own Mattress + Jay Hybrid	Continuous
12	71	F	Home	21.0	7	MS	Unstageable	IT	Powered Hybrid (Air and Foam)	Healed	Dynamic Air Mattress + Turn Device	3times per day
13	81	F	Home	24.0	8	Not recorded	Unstageable	Spine	Powered Hybrid (Air and Foam)	Healing Cat 4	Dynamic Air Mattress + Turn Device	4 times per day
15	56	M	Home	37.9	5	SCI	Unstageable	Buttock	Static Foam Mattress	Healed	Air Floatation	2 times per day
16	82	F	Residential Home	17.3	7	Dementia	Healing Cat 4	Sacrum	Powered hybrid (Air and Foam)	Healing Cat 4	Dynamic Air Mattress	Continuous
17	43	M	Home	19.6	7	Cerebral Palsy	Cat 3	Hip	Own Mattress	Healed	Air Floatation	Continuous
18	95	M	Home	23.1	7	SCI	Cat 2	Buttock	Static Foam Mattress	Cat 2	Air Floatation	2 times per day
19	68	F	Home	26.5	7	MS	Healed	N/A	Dynamic Air Mattress	Intact	Powered hybrid (Air and Foam)	4 times per day
22	65	M	Home	23.1	7	SCI	Cat 2	IT	Powered hybrid (Air and Foam)	Healed	Dynamic Air Overlay	3 times per day

Abbreviations: CVE = Celebrovascular Events; IT = Ischial Tuberosities; MS = Multiple Sclerosis; PMH = Past Medical History; SCI = Spinal Cord Injury.

### 2.4 Data Analysis

An intelligent algorithm using a custom software developed in Matlab (Mathworks, USA), which converts the CPM pressure data into indicators of posture and mobility, was used for this secondary analysis and to evaluate pre‐ and post‐ PROMISE intervention. This has been detailed in a recent paper [15], and adapted for the current analysis.

To review briefly, the algorithm used the derivative signal of combined parameters, including the centre of pressure (COP) in both planes and the contact area above a specific threshold (20 mmHg), to identify the large‐scale movements in the form of postural changes. These were defined as movements where clear evidence of changes in the spatial distribution of pressures was achieved. Movement features were estimated for each patient, involving the interval between postural changes and the duration of each static posture. Following the detection of postural changes, the COP was used to map the buttock area during each of the static postures. This was achieved by isolating a region of interest (ROI) of 47.7 × 31.8 cm^2^ (30 × 20) sensels. This area, as depicted in the clinical outcomes (Table 2), represents the region where the majority of patients had a pressure injury.

**TABLE 2:** Movement features pre‐ and post‐ PROMISE intervention, estimated from the continuous pressure data following prediction with the intelligent algorithm.

Patients No.	Pre‐ PROMISE intervention		Post‐ PROMISE intervention	
	Length of monitoring [hours]	Number of postures	Length of monitoring [hours]	Number of postures
1	14.5	17	3	2
2	16	1	14.5	1
5	9.3	3	23	8
6	9.5	3	9	4
7	15	7	15.8	2
8	20.4	6	22	12
9	23.4	9	22.5	22
10	9	5	25	10
11	51	31	23	1
12	45	5	27.5	15
13	34.5	5	24	14
15	19	5	22.5	2
16	45.3	37	40.8	8
17	22	6	34.5	13
18	35	9	11.7	1
19	13.5	6	17.3	11
22	14.5	15	15.3	5
Median	19	6	22.5	8
Range	9–45.3	4	8.7	10

**FIGURE 2:**
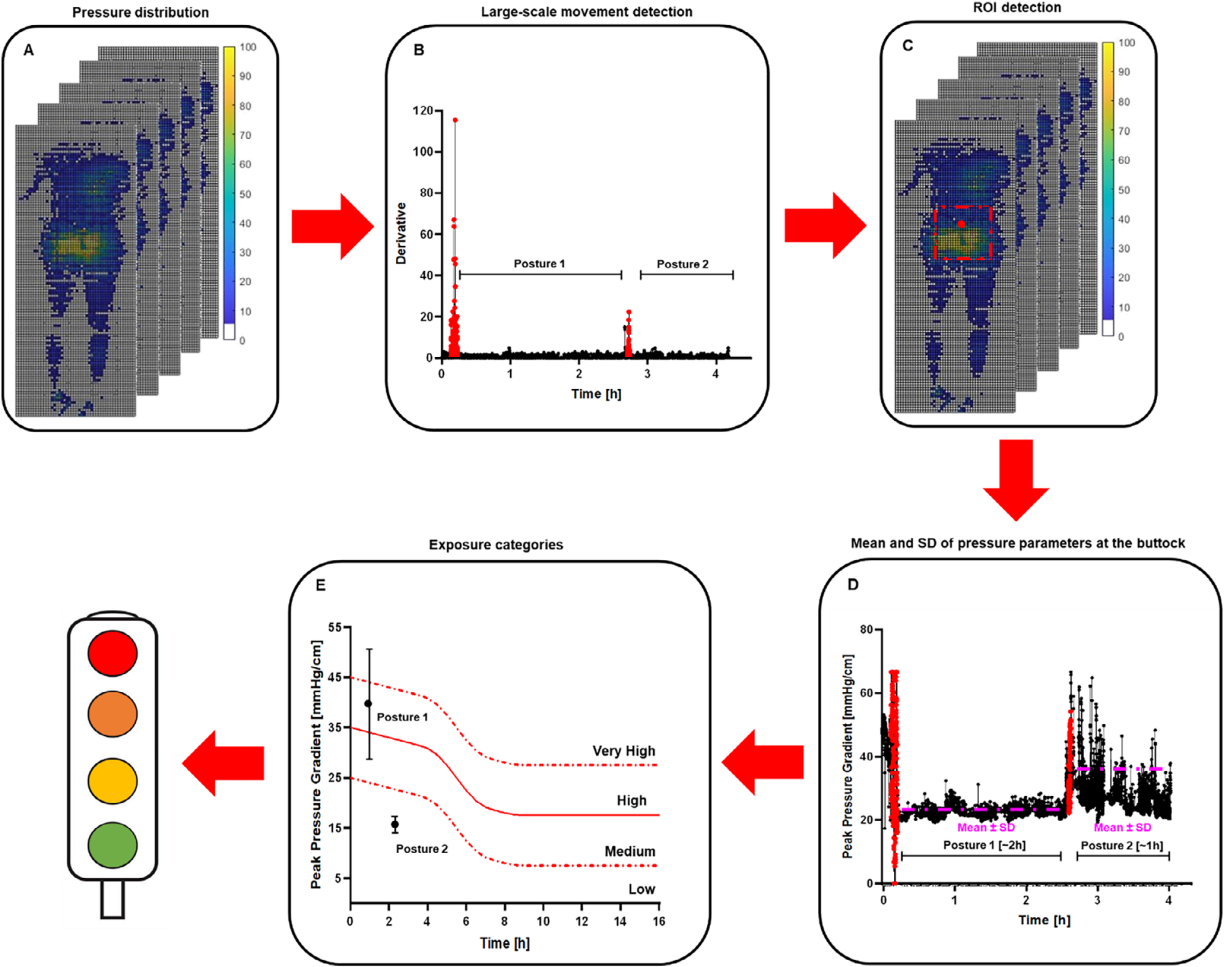
Schematic representing the adapted methodology which uses the continuous pressure data (A) to: (B) detect the large‐scale movements [changes in posture]; (C) buttock region identification through the position of the COP; (D) estimation of pressure parameters for example, peak pressure gradient, within the ROI; (E) and categories of exposure based on the pressure parameters.

For each patient, the magnitude and duration of temporal pressure signatures such as peak pressure gradient (PPG) and peak pressure index (PPI) were estimated at the buttock site during each of the static postures. To briefly review, peak pressure gradient is defined as the change in pressure between adjacent sensing cells, with a measurement unit of mmHg/cm. In the present study, it was calculated in a diagonal direction with respect to the long axis of the mat. Peak pressure index was estimated using the average of the 10 highest pressures from the ROI. Parametric descriptors of these signals were defined (mean ± standard deviation (SD)), and the resulting pressure–time relationship was then evaluated relative to sigmoid injury thresholds [21], referring to low, moderate, high, and very high pressure–time exposure. These thresholds were adapted to reflect the nature of the pressure parameters. In particular, we identified 25, 35, and 45 mmHg as starting pressures for peak pressure gradient (Figure 2E) and 70, 90, and 110 mmHg for the peak pressure index [22, 23]. Figure 2 shows a schematic representing the adapted methodology to detect posture, mobility, pressure signatures, and categories of exposure for this second analysis of the PROMISE study.

For each patient, the proportion of time in each exposure threshold as a product of the total monitoring period was calculated, pre‐ and post‐ PROMISE intervention. In addition, the frequency of movements and the percentage of time spent in a static posture for more than 2 h was also evaluated.

## 3 Results

### 3.1 Patients

Patients included 11 females and 6 males (Table 1). They ranged from 30–92 years of age and had a Body Mass Index (BMI) ranging from 15.10–46.5 kg/m^2^ (mean = 27 kg/m^2^), measured predominantly using mid‐upper arm circumference (MUAC) [24]. Levels of frailty was assessed using the Rockward Clinical Frailty Scale [25] and ranged from 5 (wheelchair users) to 8 (end of life patients). They received a variety of care arrangements, provided by both formal and informal carers. This varied from one to four visits per day for those living at home where care was frequently supplemented by live‐in or visiting relatives, to 24‐h care in both residential and nursing care home settings.

All patients received education and support related to repositioning, posture, and the correct use of the knee break mechanism to avoid sliding down the bed and causing shear damage. Interventions varied and included upgrading or downgrading of mattresses and cushions, adjustments to mattress weight settings, and tailored advice and education on postural changes and repositioning strategies. Each patient received between one and 7 CPM intervention sessions focusing on mattress related issues. Positioning aides were provided where appropriate.

Of the 17 patients, 16 had existing pressure damage prior to the intervention. Patient 19 had healed PU prior to the commencement of CPM and remained intact post intervention. The breakdown of pressure injury severity pre‐intervention was as follows: Category 2 (*n* = 8), Category 3 (*n* = 1), Category 4 (*n* = 1), Healing Category 4 (*n* = 1), and Unstageable (*n* = 5). Post‐intervention outcomes showed marked improvement: Category 2 (*n* = 1), Healing Category 4 (*n* = 1), Unstageable (*n* = 1), and Healed (*n* = 12).

### 3.2 Clinical Observations From Continuous Pressure Monitoring

Visual inspection of the CPM colour contour maps revealed an improvement of both pressure gradients and peak pressure index values between the pre‐ and post‐intervention assessments. Indeed, most patients had high perceived values pre‐intervention (12/17) compared to post‐intervention (7/17). Three patients had turning devices introduced as part of the agreed management plan, with two of them (P8 and P13) showing a decrease in the peak pressure index and gradients during this turning cycle. Of the four patients that remained unhealed (P8, P13, P16, P18) at the end of the study, only one showed an improvement post‐intervention in the pressure signatures.

### 3.3 Algorithm Derived Outcomes of Posture, Mobility and Exposure Categories

Table 2 shows the movement features pre‐ and post‐ PROMISE intervention for all 17 patients. Data revealed differing monitoring periods between patients and between interventions, with a minimum of 9 h (P10) and maximum of 45.3 h (P16) pre‐intervention (median = 19, IQR = 13) and minimum of 3 h (P1) and maximum of approximately 41 h (P16) post‐intervention (median = 22.5, IQR = 8.7). A high degree of subject variability was evident in both frequency of postural changes and number of postures adopted. The frequency (number of postural changes divided by length of monitoring period) ranged between 0.06–1.17 and between 0.06 and 0.98 movements per hour, pre‐ and post‐ PROMISE intervention, respectively, with no evident trend. Closer examination of data revealed that in pre‐ PROMISE intervention, 4 patients for example, P1, P11, P16, P22, adopted a large number of static postures (≥ 15), with these ranging between 1–9 static postures in the remainder of the patients. It was interesting to observe that these patients were all monitored on an air mattress, either static (P11) or dynamic. By contrast, post‐ PROMISE intervention data revealed a higher variability, with 7 patients adopting more than 10 static postures across the monitoring period.

**FIGURE 3:**
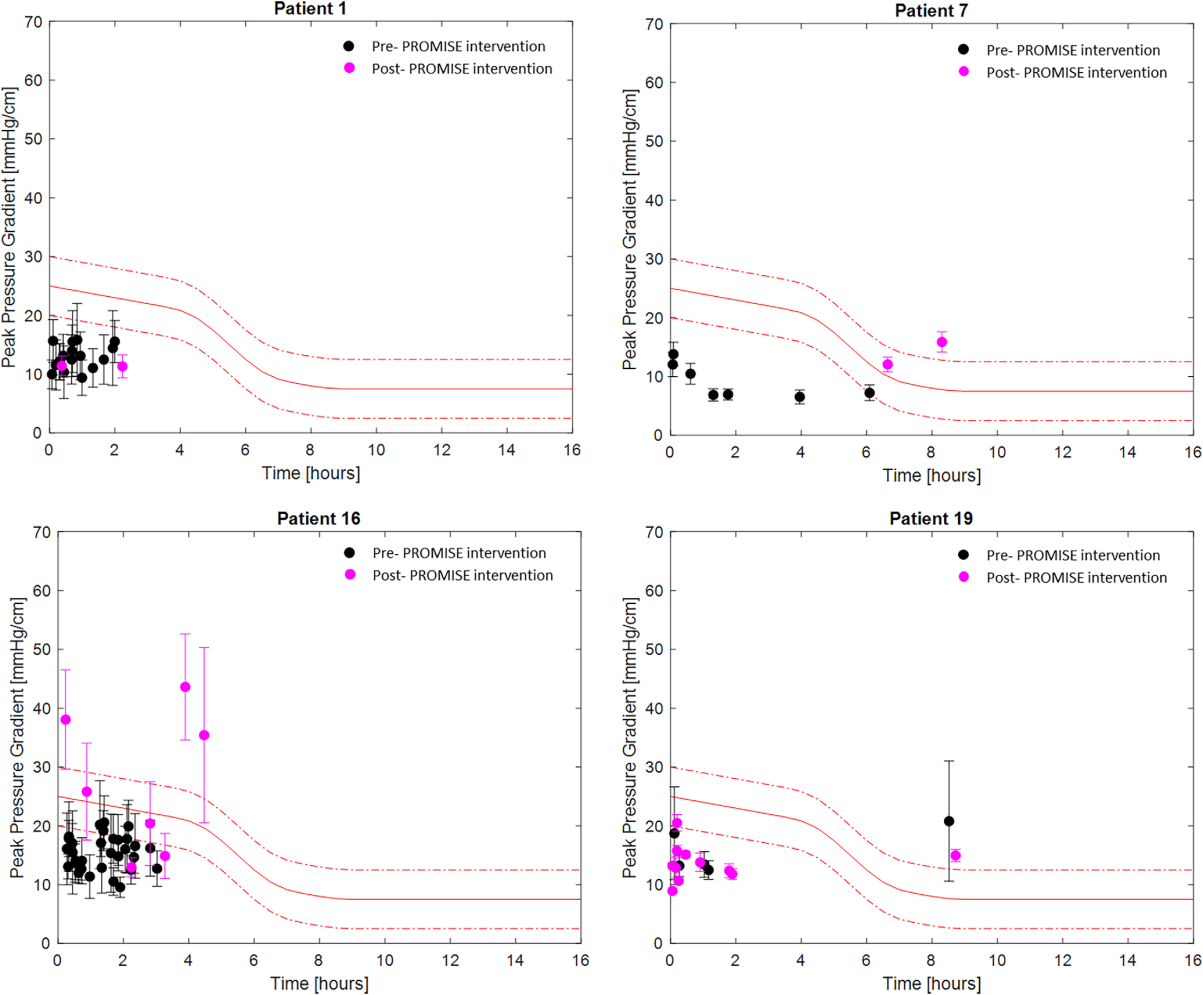
Time [hours] (x‐axis) and magnitude (y‐axis) of the mean peak pressure gradient (SD represented with the error bars) during each of the static postures, for patients P1, P7, P10, and P19, pre‐PROMISE (black markers) and post‐PROMISE (magenta markers). In red are represented the three arbitrary injury thresholds for the peak pressure gradient, based on the pressure–time sigmoid relationship.

**FIGURE 4:**
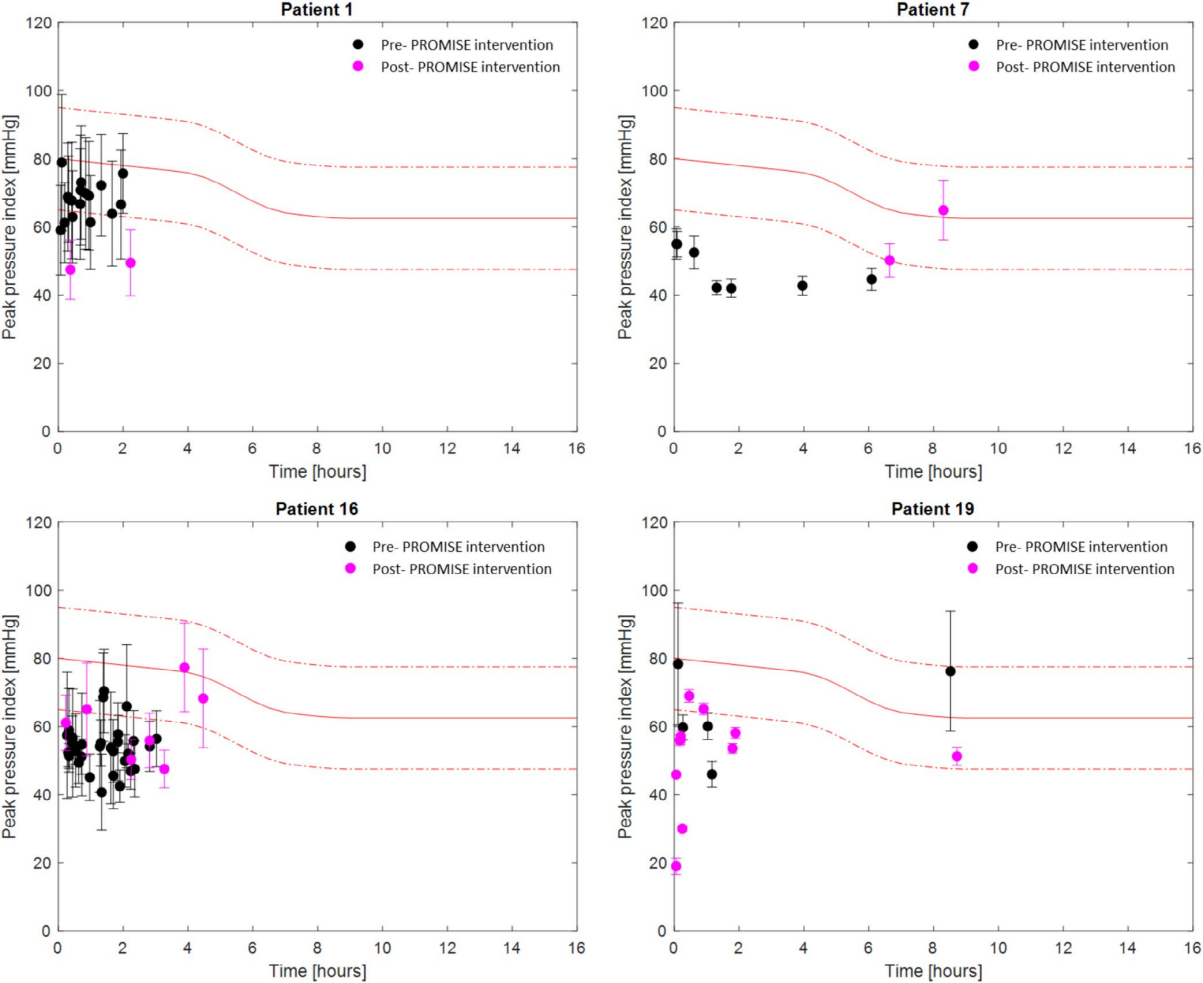
Time [hours] (x‐axis) and magnitude (y‐axis) of the mean peak pressure index (SD represented with the error bars) during each of the static postures, for patients P1, P7, P10, and P19, pre‐PROMISE (black markers) and post‐PROMISE (magenta markers). In red are represented the three arbitrary injury thresholds for the peak pressure index, based on the pressure–time sigmoid relationship.

Figure 3 shows the duration (x‐axis) and magnitude (y‐axis) of the mean peak pressure gradient (SD represented with the error bars) during each of the static postures for 4 patients, who were purposely selected to represent a range of patients with high and low mobility levels and high and low pressure exposure levels, with black and magenta markers representing pre‐ and post‐PROMISE intervention respectively. The counterparts' peak pressure index are shown in Figure 4.

Data revealed that the pressure–time profiles varied across patients and differed between interventions. As an example, pre‐PROMISE intervention P16 showed a relatively large number of static postures sustained for a relatively short period of time (< 3 h), with postures sustaining a mean peak pressure gradient and index below 20 mmHg/cm and 80 mmHg, respectively, and a high standard deviation as depicted by the error bars. Following PROMISE intervention, the number of static postures reduced, with a two‐fold increase in the sustained peak pressure gradient and index, and a few postures sustained for longer (~4 h). By contrast, P19 showed a similar trend in both duration and magnitude of pressures between pre‐ and post‐PROMISE intervention, as opposed to P7 who moved less and sustained higher pressures following PROMISE intervention.

The relationships between duration and magnitude of both mean peak pressure gradient and gradient are shown for all patients in the Supporting Information.

**FIGURE 5:**
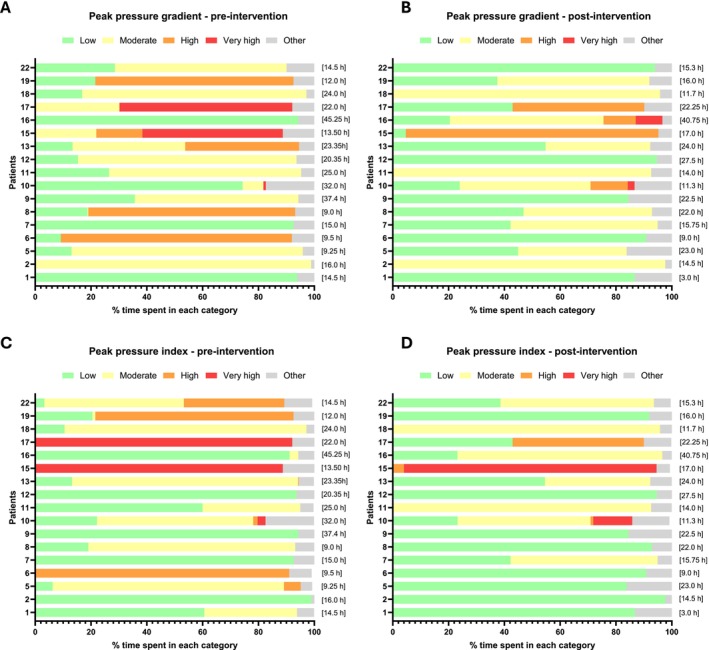
Percentage of monitoring period (x‐axis) in each exposure category (green = low, yellow = moderate, orange = high, red = very high) for all 17 patients. A and B are relative to the peak pressure gradient pre‐ (A) and post (B) PROMISE intervention, C and D are relative to the peak pressure index pre‐ (C) and post (D) PROMISE intervention. In brackets are indicated the recording period.

Figure 5 shows for all patients the percentage of time spent in the low, moderate, high, and very high pressure‐time exposure categories, pre‐ and post‐PROMISE intervention, with respect to duration and magnitude of peak pressure gradient (A and B) and peak pressure index (C and D).

It is apparent that there was a trend in reduced exposure categories from pre‐ to post‐PROMISE intervention, as depicted by a shift to more moderate to low pressure‐time exposures in the latter for both peak pressure gradient and peak pressure index. However, comparison between pre‐ and post was subject and pressure signatures dependent. For example, the majority of the patients for example, P1, P2, P5, P7, P9, P10, P11, P12, P13, P16, P18 revealed mobility and pressure signatures falling in the green and yellow categories for > 80% of their time pre‐ and post‐ intervention, for both peak pressure gradient and index. Whilst some did not show any change in the exposure category between pre‐ and post‐ intervention for example P1, P2, some showed both a shift from yellow to green for example, P5, and from green to yellow for example P16.

By contrast, some patients for example, P6, P15, P17 spent most of their time in the ‘at risk’ categories, with a reduced exposure from pre‐ to post‐ intervention.

## 4 Discussion

The secondary analysis was conducted following a QI project ‘PROMISE’ where individuals were assessed whilst in bed using a continuous pressure monitoring (CPM) technology (ForeSitePT, Xsensor, Canada) [10]. The present study aimed to evaluate whether an intelligent algorithm could detect changes in posture, mobility and pressure exposure in a cohort of community patients with pressure injuries pre‐ and post‐ the CPM informed intervention. The results revealed that in a cohort of patients there were periods of prolonged static postures in bed which exposed individuals to high peak pressure gradient and peak pressure index values. Following an intervention using the CPM technology, the risk status of prolonged static postures and high pressures reduced in several patients but also increased for some individuals. Following the PROMISE intervention, a number of patients' PU healed, which may have resulted in a combination of interventions in the bed and chair, improved patients awareness and shared decision making which requires future analysis.

The cohort assessed in the present study consisted of individuals with pressure injuries, who had mobility restrictions and high levels of frailty (Table 1). This is reflective of other studies which have characterised individuals with pressure injuries in the community [26]. The patients were provided with a range of support surfaces to manage their pressure injuries, which in many cases were changed following assessments with continuous pressure monitoring (Figure 1). This aligns with how pressure systems have been historically used, where different mattress or cushion devices could be selected based on pressure redistribution characteristics. This, however, has always been limited to short term assessments of the pressure distributions [27]. By contrast, our approach of monitoring provides a novel evaluation through intelligent algorithms [14, 28, 29], where mattress performance was assessed over time and with respect to different individuals' postures, creating a more holistic approach.

Results revealed a high degree of variability in mobility status, including individuals only moving once over a 16‐h period, to those moving more than hourly over the course of monitoring (Table 2). This reflects results from previous studies which have employed a variety of methods to monitor bed mobility [30], and those using CPM as a surrogate for movement [9]. In addition, the analysis revealed that for some postures the interface pressures in the form of peak pressure gradient and peak pressure index were low, as opposed to other postures with higher pressure exposures, thus creating risk to tissue health (Figures 3, 4, 5). Our study represents one of the few which have described these pressure signatures over prolonged periods [9, 15]. A few studies have evaluated the use of CPM in hospital settings using different pressure metrics such as average pressure, which may be less sensitive in evaluating at risk tissue sites [12].

It is of note that some patients presented with less mobility post‐PROMISE intervention, with some of them associated with higher pressure signatures. This may have been due to an increase in comfort level on the new support surface, which enabled prolonged positions in bed. Some posture may also have been held for prolonged periods to enable activities of daily living for example, eating, sitting, social engagement, for example, high sitting postures. In addition, interpretation of the pressure data is subject specific. Indeed, patients had a range of postural needs, which predisposed them to higher pressures over the buttock area. A reduction in pressures was not directly related to comfort; indeed, clinical decisions were made in partnership with the patient and carer with the only aim of improving their quality of life.

### 4.1 Limitations

Analysis was carried out on the time spent in bed, and seated data were not analysed at this time. However, three patients remained on bed rest (P8, 9 and 16), and the remaining patients were sitting out for varying lengths of time. This study is a secondary data analysis from a QI project, where interventions were not standardised or controlled, providing thus personalised interventions; these can be found in Appendix A. As a result, any changes in mobility and pressure exposure are a composite of clinical decision making, patient choice and access to care. In addition, the monitoring period varied between individuals and interventions, limiting thus the comparisons. Further evaluation is indeed required to determine how the CPM technology and associated algorithms can support clinical decision making and patient understanding of pressure injury risk. It was also possible that there were some interferences in detecting posture and mobility caused by active support surface systems, which may have increased the amount of posture and mobility events detected by the algorithm. The fixed ROI used to isolate the buttock area may also have limited the estimation of the pressure signatures; further implementation is required to isolate and recognise ROIs in a more intelligent manner. In addition, the thresholds selected for the pressure exposure were arbitrary, and further refinement of patients specific and pressure signatures thresholds is required to better reflect the variability in tissue vulnerability observed in different patient cohorts.

We were also not able to evaluate the effects on interface pressures caused by shear forces during manual handling techniques, hoist and stand‐aid transfers, slide board transfers, or shearing caused due to lack of knee break use.

### 4.2 Clinical Implications

From the PROMISE study, it emerged that continuous pressure monitoring informed and supported shared decision‐making in changing support surface settings and their selection, posture management, and empowered patient and carer understanding of pressure injury risk. Our secondary analysis evaluated whether the PROMISE intervention had an impact on mobility and pressure exposure, by the means of an intelligent algorithm and the novel use of the sigmoid risk stratification. This has shown potential to stratify levels of risk in community patients and sensitivity to change pre‐ and post‐interventions. Future research is needed to evaluate the combined use of CPM and algorithms to support pressure injury prevention and treatment in patients both within hospital and in the community setting. To ensure that implementation is successful, key human factor evaluations are required to evaluate the training needs, user interface and information sharing the CPM system can provide.

## 5 Conclusion

This study used an intelligent algorithm based on the pressure–time sigmoid relationship to evaluate mobility status and pressure exposure in a cohort of community patients with pressure injuries. CPM during PROMISE informed interventions including support surface change, posture management and patient education. Our novel approach provided the means to stratify risk, using a combination of pressure and time exposure. Further research is needed to establish its clinical and cost effectiveness to act as an adjunct to clinical and shared decision‐making for pressure injury prevention and treatment.

## Funding

The QI improvement project was supported by the Health Foundation Scaling Up Grant (509449). The secondary analysis was supported by the Engineering and Physical Sciences Research Council (EPSRC) as part of the project ‘Intelligent Sensing to Promote Self‐management of Posture and Mobility in Community dwelling Individuals’ [EP/W031558/1].

## Ethics Statement

The secondary analysis of the QI PROMISE project obtained institutional ethical approval (ERGO 80625.A1).

## Consent

All patients gave their consent to participate in the PROMISE project.

## Conflicts of Interest

The authors declare no conflicts of interest.

## Supporting information


**Appendix A** Summary of the personalised interventions during PROMISE.

## Data Availability

The data that support the findings of this study are available from the corresponding author upon reasonable request.
