# Supplementary material for: Exploring Continuous Pressure Monitoring to Inform Decisions for Pressure Injuries in the Community: Secondary Analysis Using a Mobility and Pressure Exposure Algorithm
Source: Int Wound J. 2026 Apr 12;23(4):e70896. doi: 10.1111/iwj.70896 (PMC13070666; doi:10.1111/iwj.70896)
Supplement: Supplementary file 1 — Appendix A Summary of the personalised interventions during PROMISE. [file IWJ-23-e70896-s001.docx]

**Appendix A:** Summary of the personalised interventions during PROMISE.

| **Patient ID** | **Number of CPM evaluations for mattress (M) Cushion (C)** | **Mattress (M) and Cushion (C) Intervention** | **Postural Intervention** | **Education Received** |
| --- | --- | --- | --- | --- |
| 1 | 2M +1C | M weight setting reduced | Static air overlay cushion. | Yes |
| 2 | 2M +2C | M and C upgraded | Referred to community rehab for support | Manual handling education to carer |
| 5 | 2M +3C | M & C Upgraded | J/V with OT | Yes |
| 6 | 2M +2C | M & C Upgraded | Slight lean to the left. Sits on side of bed. High pressures in W/C - referral to W/C services to review cushion – Pt not using new cushion. | / |
| 7 | 7M + 2C | M & C upgraded | Limited sensation  AHP - Not able to change position at night – sleep system provided | Yes |
| 8 | 1M | Turning device | Contracted legs- unable to lie on back – bed rest | Carer education |
| 9 | 3M | M upgraded | Sleeps upright in bed - | Patient education |
| 10 | 2M + 2C | M upgraded | Sleeps on front due to spina bifida | Patient/carer education on use of turning device |
| 11 | 2M + 2C | Patients own overlay removed damage | Sits up in bed in the morning | Advised to use knee break to stop shearing |
| 12 | 2M + 1C | M & C upgraded – Turning device | Contractures – referral to neuro physio | Education given to stop lying on left side and bed rest if possible |
| 13 | 2M | M Upgraded – Turning device | Leans to left – AHP referral – 24 hr bed rest. | Advice given on times of turning device and use of knee break |
| 15 | 5M + 6C | M upgraded | Postural difficulties – joint visit with w/c services | Education and advice given to reduce sitting time |
| 16 | 3M | M upgraded | Limited sensation - contractures | Education on repositioning shared with carers and daughter |
| 17 | 5M +4C | M upgraded | Curvature of spin and bulging chest due to cerebral palsy –  AHP referral | Education and advice to carers |
| 18 | 1M + 1C | M upgraded | AHP referral | Advice and Education to increase repositioning and use slide transfers |
| 19 | 2M + 3C | M changed to air floatation from dynamic | J/V with w/c services | Advised to use w/c in recline position – now able to sit out for 6 hrs daily |
| 22 | 2M +1C | M Upgraded | / | / |

**=Mattress C=Cushion W/C=Wheelchair J/V=Joint visit AHP=Allied Health Professionals**
